# False-negative malaria rapid diagnostic test results and their impact on community-based malaria surveys in sub-Saharan Africa

**DOI:** 10.1136/bmjgh-2019-001582

**Published:** 2019-07-29

**Authors:** Oliver J Watson, Kelsey Marie Sumner, Mark Janko, Varun Goel, Peter Winskill, Hannah C Slater, Azra Ghani, Steven R Meshnick, Jonathan B Parr

**Affiliations:** 1 MRC Centre for Global Infectious Disease Analysis, Imperial College London, London, UK; 2 Department of Epidemiology, Gillings School of Global Public Health, University of North Carolina, Chapel Hill, North Carolina, USA; 3 Global Health Institute, Duke University, Durham, North Carolina, USA; 4 Department of Geography, University of North Carolina, Chapel Hill, North Carolina, USA; 5 Division of Infectious Diseases, Department of Medicine, University of North Carolina, Chapel Hill, North Carolina, USA

**Keywords:** malaria diagnosis, rapid diagnostic tests, RDTs, *pfhrp2*, pfhrp2 deletion, *Plasmodium falciparum*, mathematical modelling, mapping

## Abstract

Surveillance and diagnosis of *Plasmodium falciparum* malaria relies predominantly on rapid diagnostic tests (RDT). However, false-negative (FN) RDT results are known to occur for a variety of reasons, including operator error, poor storage conditions, *pfhrp2/3* gene deletions, poor performance of specific RDT brands and lots, and low-parasite density infections. We used RDT and microscopy results from 85 000 children enrolled in Demographic Health Surveys and Malaria Indicator Surveys from 2009 to 2015 across 19 countries to explore the distribution of and risk factors for FN-RDTs in sub-Saharan Africa, where malaria’s impact is greatest. We sought to (1) identify spatial and demographic patterns of FN-RDT results, defined as a negative RDT but positive gold standard microscopy test, and (2) estimate the percentage of infections missed within community-based malaria surveys due to FN-RDT results. Across all studies, 19.9% (95% CI 19.0% to 20.9%) of microscopy-positive subjects were negative by RDT. The distribution of FN-RDT results was spatially heterogeneous. The variance in FN-RDT results was best explained by the prevalence of malaria, with an increase in FN-RDT results observed at lower transmission intensities, among younger subjects, and in urban areas. The observed proportion of FN-RDT results was not predicted by differences in RDT brand or lot performance alone. These findings characterise how the probability of detection by RDTs varies in different transmission settings and emphasise the need for careful interpretation of prevalence estimates based on surveys employing RDTs alone. Further studies are needed to characterise the cost-effectiveness of improved malaria diagnostics (eg, PCR or highly sensitive RDTs) in community-based surveys, especially in regions of low transmission intensity or high urbanicity.

**Key questions**

**What is already known?**

- False-negative rapid diagnostic test (RDT) results can be caused by a number of factors.
- Microscopy is the traditional diagnostic gold standard for malaria. RDT-negative but microscopy-positive results can occur due to operator error, poor storage conditions, *Plasmodium falciparum* histidine-rich protein 2 and 3 gene deletions, poor performance of specific RDT brands and lots, and low-parasite density infections.

**What are the new findings?**

- Using national surveys from 19 countries in sub-Saharan Africa between 2009 and 2015 in combination with data from standardised RDT product testing, we find that false-negative RDT results (RDT negative but microscopy positive) within community surveys are not predicted by lot or brand alone.
- A significant increase in false-negative RDT results is observed at lower transmission intensities, among younger children, and within urban areas.

**What do the new findings imply?**

- The increased frequency of false-negative RDT results in low transmission and urban settings implies that targeted use of next-generation, higher sensitivity diagnostics may be appropriate in these settings.

## Introduction

Malaria kills thousands of children in sub-Saharan Africa each year, with an estimated 266 000 malaria deaths among children under 5 years of age in 2017.^1^ Rapid diagnostic tests (RDT) are the primary mode of malaria diagnosis in Africa, accounting for 75% of diagnostic testing for suspected malaria in public facilities in 2017.^1^ Most commonly used RDTs in Africa identify malaria infection by detecting the presence of *Plasmodium falciparum* histidine-rich protein 2 (PfHRP2), a *P. falciparum*-specific antigen, in the blood.^2^ RDTs were initially designed for clinical diagnosis; however, they are also used for community-based surveillance as they permit rapid diagnosis in field settings, are easier to implement than microscopy and have been shown to have comparable detection capabilities in the field.^3^

RDT performance, however, can vary. It is well known that false-positive results occur because the PfHRP2 antigen lingers in the bloodstream after an infection is cleared. Treated patients may maintain detectable levels of HRP2 for months after parasite clearance, which can lead to overtreatment and the misdiagnosis of the true cause of symptoms.^4^ False-negative (FN) results due to RDT failure, RDT misuse or misinterpretation of test bands can also result in discordance between the outcome of diagnosis by microscopy and RDT. These FN-RDT results, which we will define as RDT-negative but microscopy-positive results, are well documented and are of particular concern because they can lead to the misdiagnosis of malaria infections.

There are several causes of FN-RDT results. First, the parasite density may be below the RDT’s limit of detection (LOD), typically in the range of 200 parasites/µL.^5^ RDT LODs widely vary, sometimes even within the same country as has been observed in Angola.^6^ Second, non-*P. falciparum* malaria is not detected by commonly used PfHRP2-based RDTs.^7^ Third, improper RDT storage, including prolonged exposure to hot or humid conditions, can impair RDT performance.^8^ Fourth, operator error can occur if the RDT test bands are misinterpreted or if the result is read before or after the recommended incubation period.^9^ Finally, lot-to-lot variation can influence RDT performance.^10^

One cause of FN-RDT results that has recently garnered much attention is deletion of the *pfhrp2/3* genes. These deletions have been reported in multiple locations, first in South America and now in Asia and Africa.^11^ Parasites bearing these gene deletions are not detected by PfHRP2-based RDTs,^12^ leading to the prediction that the continued use of RDTs that detect PfHRP2 alone will select for parasites with *pfhrp2/3* deletions.^13^ The geographic distribution of these deletions, however, is not known, although increasing reports indicate that they are widely distributed, and have been observed in 11 African nations (online supplementary table S1).^14^ The WHO has produced a Malaria Threats Map, which catalogues and visualises the distribution of studies observing *pfhrp2/*3, as well as insecticide and antimalarial resistance.^15^

10.1136/bmjgh-2019-001582.supp1 Supplementary data

In this study, we use household survey data collected during Demographic and Health Surveys from sub-Saharan Africa. In these surveys, both RDTs and microscopy were performed on 85 000 children under 5 years of age, which provided an opportunity to calculate malaria prevalence and the proportion of FN-RDT results (RDT negative, microscopy positive) as determined by quality-controlled microscopy. Our results show that FN-RDT results are widespread, vary dramatically by geography and may have significant impacts on malaria surveys and control efforts.

## Methods

### Data set description

This study used a data set compiled from Malaria Indicator Surveys (MIS) and Demographic Health Surveys (DHS) conducted by the DHS Program^16^ and extracted using rdhs.^17^ Performed in endemic malaria regions, MIS and DHS are rigorous, cross-sectional household surveys performed using a multistage cluster-randomised sampling design. Children aged 0–60 months who were members of chosen households or had stayed in the household the previous night were eligible for the survey. Basic demographic information and malaria diagnostic results were collected for each participant aged 60 months or younger. Microscopy and RDT testing was performed for all eligible children for whom consent was given for malaria testing according to DHS protocol.^18^ Microscopy results were read by two independent technicians, discrepancies resolved by a third reader and rigorous quality control performed by an external quality control laboratory for 5%–10% of all slides read.^19^ All RDT assays were performed and interpreted by health technicians trained according to the RDT manufacturer’s instructions and assisted by the field team nurse. Technicians were provided with a timer to ensure the correct time had passed before reading the RDT test bands.^20^

For this analysis, we compiled the most recent MIS and DHS surveys that included DHS cluster Global Positioning System coordinates from 2009 onwards in sub-Saharan Africa and included both microscopy and RDT testing results for all eligible children. The main outcome, an FN-RDT result, was measured at the individual level and defined as a positive gold standard microscopy test result for any *Plasmodium* species but negative RDT result. The percentage of FN-RDT results was calculated as the total number of FN-RDT results in a country, first-level administrative region (eg, province)—hereafter, administrative regions are always referred to at the first level—or DHS sampling cluster divided by the total number of microscopy-positive results expressed as a percentage. Sample weights provided by the DHS were incorporated to account for complex survey design when calculating the percentage of FN-RDT results within administrative regions and countries.

### Expected percentage of FN-RDT results as determined by the WHO Product Testing Programme

The WHO Product Testing Programme monitors the quality and effectiveness of RDTs distributed worldwide.^21^ The programme evaluates RDT performance across brands using standardised panels of parasites and disseminates the results. Product lot testing results for the RDTs used in the DHS and MIS were publicly available for surveys conducted in 19 countries between 2009 and 2015: Angola, Benin, Burkina Faso, Burundi, the Democratic Republic of the Congo, Côte d’Ivoire, Guinea, Kenya, Liberia, Madagascar, Malawi, Mali, Mozambique, Nigeria, Rwanda, Senegal, Tanzania, Togo and Uganda. The provided data were used to estimate the performance of each RDT brand used during the DHS/MIS studies. We adjusted the observed country-level FN-RDT results in our data set by subtracting the expected percentage of FN-RDT results based on the WHO Product Testing Programme’s Panel Detection Score (PDS), using the PDS at 200 parasites/μL for each RDT brand and pairing lot testing results and study RDTs based on the most proximate RDT expiration date. The PDS is a conservative measure of how well an RDT is expected to perform in the field and is not a true measure of clinical sensitivity.^10^ Although the PDS will not perfectly estimate an RDT’s true performance in field settings, it provides an approximation of how often an RDT would fail to detect microscopy-positive infection in the field. We calculated the expected and adjusted percentage of FN-RDT results from the product testing results as follows:

­

$${FN\text{-}RDT}_{expected}=100-PDS$$

$${FN\text{-}RDT}_{adjusted}={FN\text{-}RDT}_{observed}-{FN\text{-}RDT}_{expected}$$

We assessed the appropriateness of using the 200 parasites/μL PDS results in our expected FN-RDT calculations by examining the distributions of parasite densities among microscopy-positive subjects enrolled in published, cross-sectional studies from countries included in this study (see online supplementary methods).^22^

### Spatial clustering analysis

Because the geographic distribution of FN-RDT results is unknown, we mapped DHS cluster-level FN-RDT results within each of the 19 countries. To evaluate whether FN-RDT values exhibited a spatially clustered pattern, we tested for spatial autocorrelation using the Global Moran’s I measure in GeoDa.^23^ Spatial autocorrelation was assessed across each of the 19 countries. The distance radius used to determine neighbouring values for spatial clustering for each country was determined by inspection of individual country FN-RDT correlograms.

### Hierarchical model and subanalysis of significant variables

To explore the risk factors for FN-RDT results, we fit a Bayesian mixed effect logistic regression model, where the outcome is whether or not each child under 5 years of age had an FN-RDT result. Potential risk factors included: individual age, sex, survey year, cluster-level malaria prevalence, cluster size, RDT brand, urban residence and the seasonality at the time of sample collection estimated using previously fitted seasonality curves.^24^ Country was not chosen to be a fixed effect due to the resultant rank deficiencies that would form in the model matrix, resulting from only having data from one RDT brand per country. However, country was included as a random effect with both a random slope and intercept with respect to malaria prevalence.

The model was fit to the individual-level data within a Bayesian framework using Markov chain Monte Carlo techniques. We used uninformative parameter expanded priors for the variance-covariance matrices of the random effects. All models were run for 100 000 iterations, with a burn-in of 10 000 iterations. Convergence of chains was confirmed by both assessing covariate effective sample sizes to be greater than 1000 for all parameters and visually checking for convergence between multiple chains. The posterior mean and 95% highest posterior density interval for the log ORs of the fixed effects is presented.

### Malaria infections missed during malaria surveys due to RDT-negative results

Using an individual-based malaria transmission model,^25^ we incorporated the estimated proportion of FN-RDT results for each administrative region to estimate the percentage of malaria infections that would not be detected during community-based malaria surveys using RDTs, compared with microscopy. An overview of the main model inputs and methodology is described briefly, with full mathematical details, parameter values and the precise simulations as previously described.^26^

In overview, the transmission model considers individuals to exist in one of six states: susceptible (S), clinical disease (D), clinically diseased and receiving treatment (T), in a protective state of prophylaxis (P), subpatent asymptomatic infection (U) and potentially patent asymptomatic infection (A). The probability that an individual will be detected by microscopy in a survey, *p(detection*), is dependent on their infection state, which defines the range of parasitaemia an individual may possess. In addition, an individual’s age and acquired immunity will also alter *p(detection*) in the model (online supplementary methods). In order to estimate *p(detection*) for each administrative region, simulations fitted to mapped estimates of malaria prevalence from 2000 to 2015 were conducted.^27^ For each region we incorporated the historical scale-up of vector-based interventions (estimated using data collated for the World Malaria Report)^28^ and the treatment coverage from previously modelled estimates using DHS and Multiple Indicator Cluster Survey (MICS) surveys for each administrative region.^29^ Seasonality for each region was incorporated in the form of annually fluctuating seasonal curves fitted to daily rainfall data from 2002 to 2009.^30^ Lastly, the at-risk population was delimited using previously defined spatial limits for *P. falciparum* transmission.^31^ These limits were combined with population estimates from the Gridded Population of the World data set,^32^ after being adjusted to account for the United Nations’ estimates of country populations, to define the total population size at risk of malaria.

For a given individual *i* in administrative region *j*, the probability that s/he would yield an FN-RDT result (negative by RDT but positive by microscopy) is given by:

$p{\left(detection\right)}_{i}*FN\text{-}RDT_{j}$, where *FN-RDT_j_* is the proportion of FN-RDT results in administrative region *j* and *p(detection)_i_* is the individual’s probability of being detected by microscopy. The percentage of missed infections is subsequently given by:

$\%MissedInfections=\frac{\sum_{j=1}^{m}\left(\sum_{i=1}^{n_{j}}p{\left(detection\right)}_{i}*\left(1-FN-RDT_{j}\right)\right)}{\sum_{j=1}^{m}\left(\sum_{i=1}^{n_{j}}p{\left(detection\right)}_{i}\right)}$

where *n_j_* is the total number of individuals in administrative region *j* and *m* is the total number of administrative regions. For each country estimate, the 95% CI was calculated, using the corresponding CI for the sample weighted estimate of FN-RDT results for each administrative region.

### Patient and public involvement

This research was conducted without patient involvement, using deidentified national survey data.

## Results

The analysis included exactly 85 000 participants aged 0–60 months after exclusion of observations with missing RDT or microscopy data. Participants were chosen from 19 countries in sub-Saharan Africa between 2009 and 2015, with the most samples from the Democratic Republic of the Congo (9.7%) and least from Malawi (2.3%). Across all studies, the mean malaria prevalence by microscopy was 24.4% and by RDT was 30.3% (online supplementary table S2). SD Bioline Malaria Ag Pf (Abbott, Abbott Park, IL, USA) was the most common RDT brand, accounting for 26.2% of RDTs used. Across all studies, the mean percentage of FN-RDT results was 19.9% (95% CI 19.0% to 20.9%), but there was substantial variation between countries.

**Figure 1:**
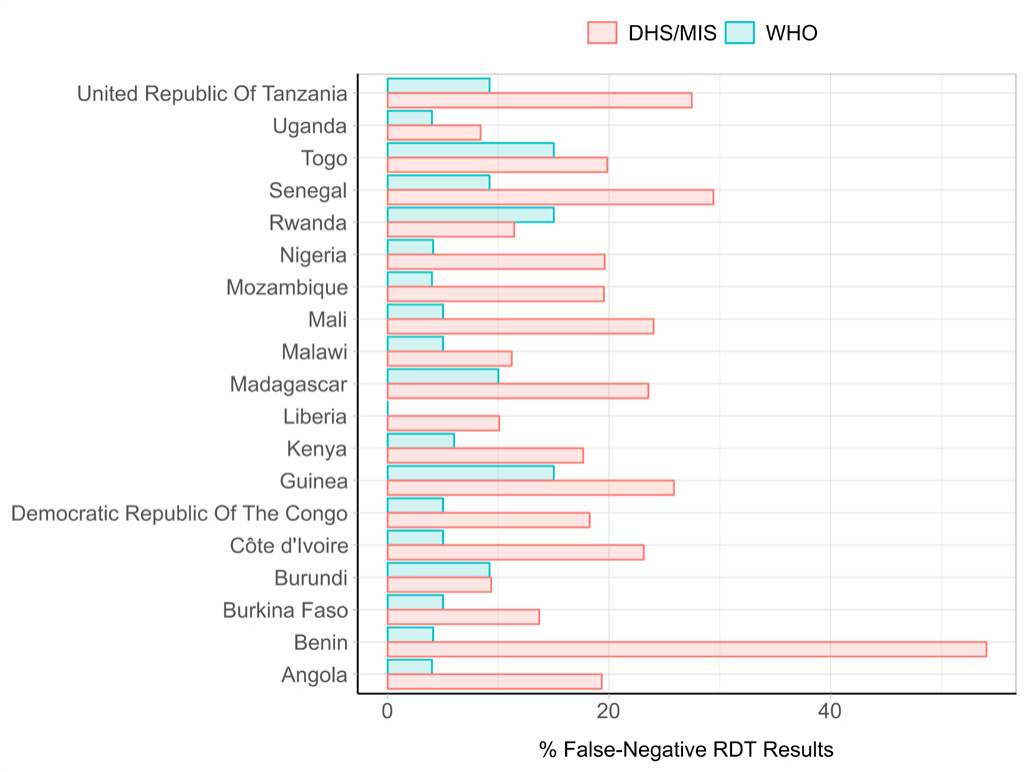
Observed percentage of false negative rapid diagnostic test (FN-RDT) results from DHS and MIS field surveys and expected results based on the WHOFoundation for Innovative New Diagnostics (FIND) lot testing. Red bars depict the weighted percentage of microscopy-positive infections that yielded a negative RDT result in the DHS/MIS surveys, and blue bars depict the expected percentage of microscopy-positive, RDT-negative infections based on a conservative estimate derived from the WHO Product Testing Panel Detection Score (PDS) at parasite densities of 200 parasites/μL. DHS, Demographic Health Surveys; MIS, Malaria Indicator Surveys.

The percentage of FN-RDT results observed in the field based on the DHS data was higher than the expected FN-RDT results based on product testing results for most DHS surveys (figure 1). Some countries like Benin had marked differences in observed FN-RDT results during the DHS survey (54.1%, 95% CI 50.0% to 58.2%) and expected FN-RDT results based on product testing of the RDT employed during the survey (4.1%). In contrast, RDT performance during the Burundi survey was similar to the performance predicted by product testing, with an observed percentage of FN-RDT results of 9.4% (6.2–12.5) compared with the expected percentage of FN-RDT results of 9.2% based on product testing. Prevalence influenced the statistical precision of the observed FN-RDT results, with the number of microscopy-positive results in each DHS cluster or country being the denominator for the observed proportion of FN-RDT results. Prevalence of infection by microscopy varied widely, with Mali having the highest malaria prevalence (51.0%, 95% CI 47.6% to 54.3%), and Rwanda having the lowest (2.3%, 95% CI 1.7% to 3.0%). Evaluation of available, published data suggested that low-parasite density infections are not likely the sole driver of the differences between the observed and expected FN-RDT prevalence. The median parasite density as determined by quantitative PCR (qPCR) observed among microscopy-positive subjects in these cross-sectional studies was above 200 p/µL (online supplementary figure S3).

**Figure 2:**
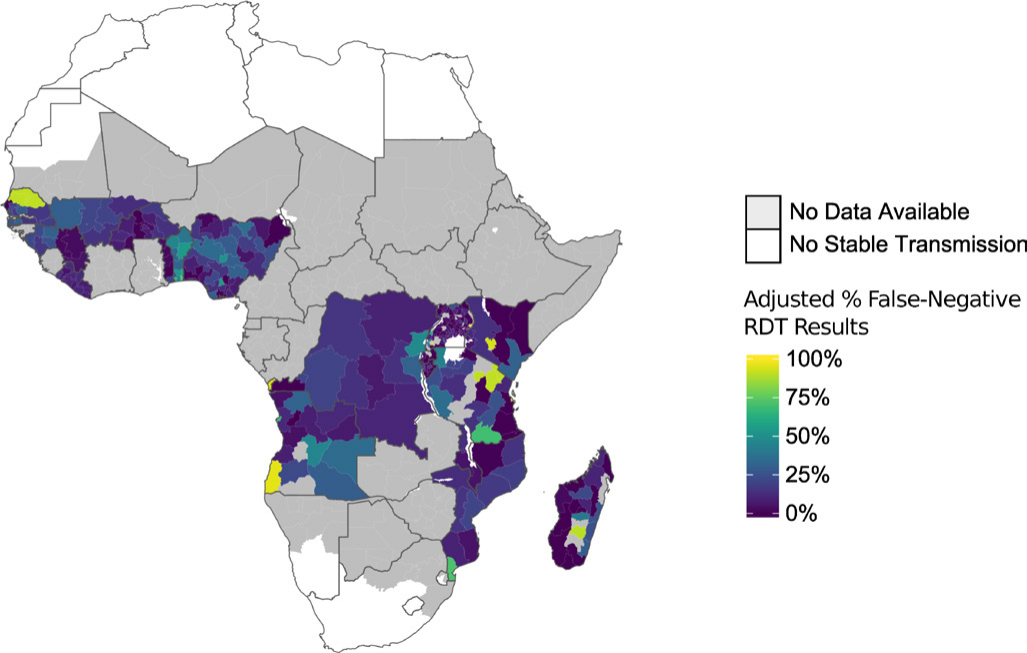
Mean adjusted percentage of false negative rapid diagnostic test (FN-RDT) results at the first administrative region. The percentage of FN-RDT results presented represents the data from the most recent Demographic Health Surveys (DHS) or Malaria Indicator Surveys (MIS) for each country for which both RDT and microscopy results were available, after adjusting for the expected percentage of FN-RDT results based on WHO product testing results.

The spatial distribution of adjusted FN-RDT results was heterogeneous across the countries studied (figure 2). At a country level, Benin had the highest adjusted percentage of FN-RDT results (50.0% (45.9–54.1)). Overall, FN-RDT results were more frequent in Western Africa than other regions (range: 4.83%–50.0%). At the DHS cluster level, FN-RDT results differed within countries, with some areas having higher frequencies of FN-RDT results than others. Global Moran’s I results suggested that there was not a strong spatially clustered pattern for FN-RDT results at the DHS cluster level for each country (online supplementary table S3). While a number of countries had statistically significant Global Moran’s I values, the values were all close to 0. Thus, these country-level analyses did not provide strong evidence of spatial autocorrelation of FN-RDT results.

**Figure 3:**
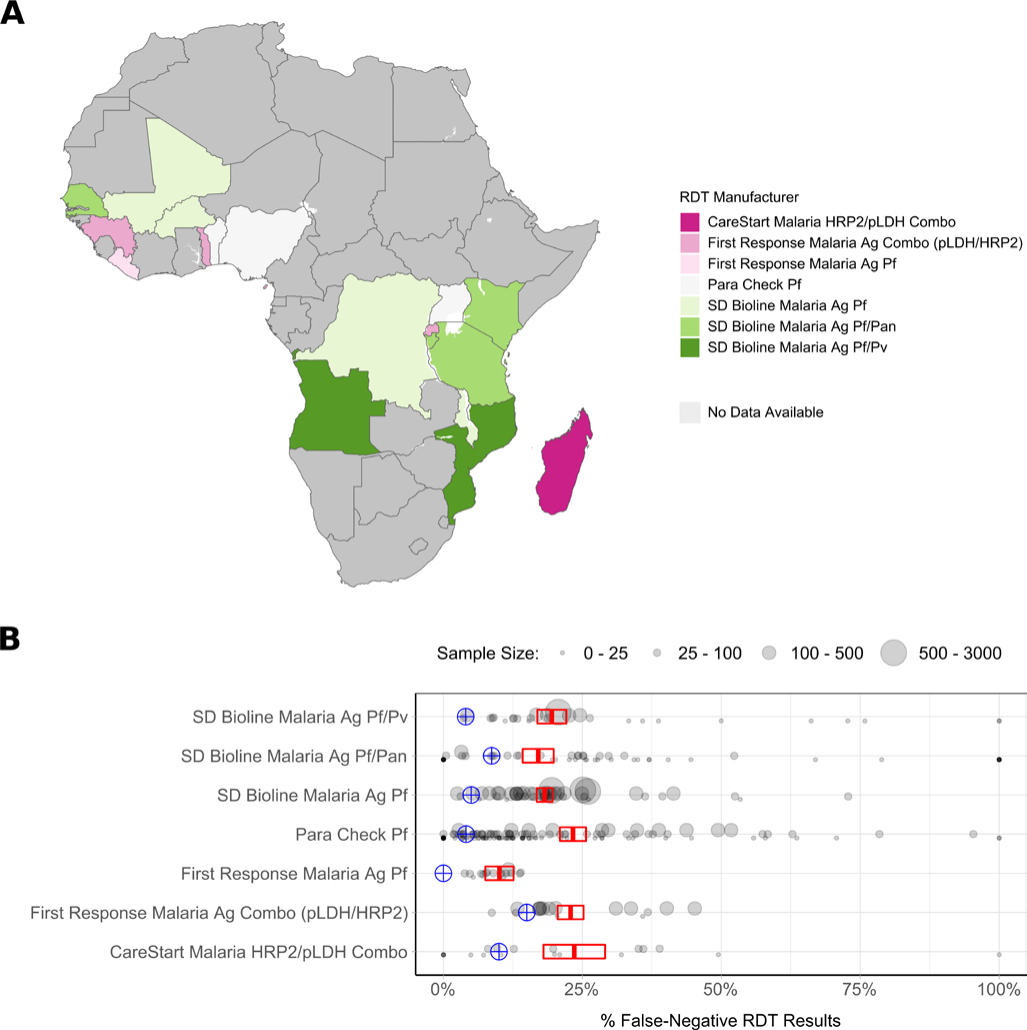
Distribution and impact of rapid diagnostic test (RDT) brand upon the observed percentage of false-negative (FN) RDT results. In (A) the RDT brand used within the Demographic Health Survey (DHS) years of interest is shown. In (B) the weighted percentage of FN-RDT results at the first administrative region is shown for each RDT brand. The sample size for each region is indicated by the point size, and the mean and 95% CI for each brand is shown in red, with the expected result based on WHO product testing shown in blue.

**Figure 4:**
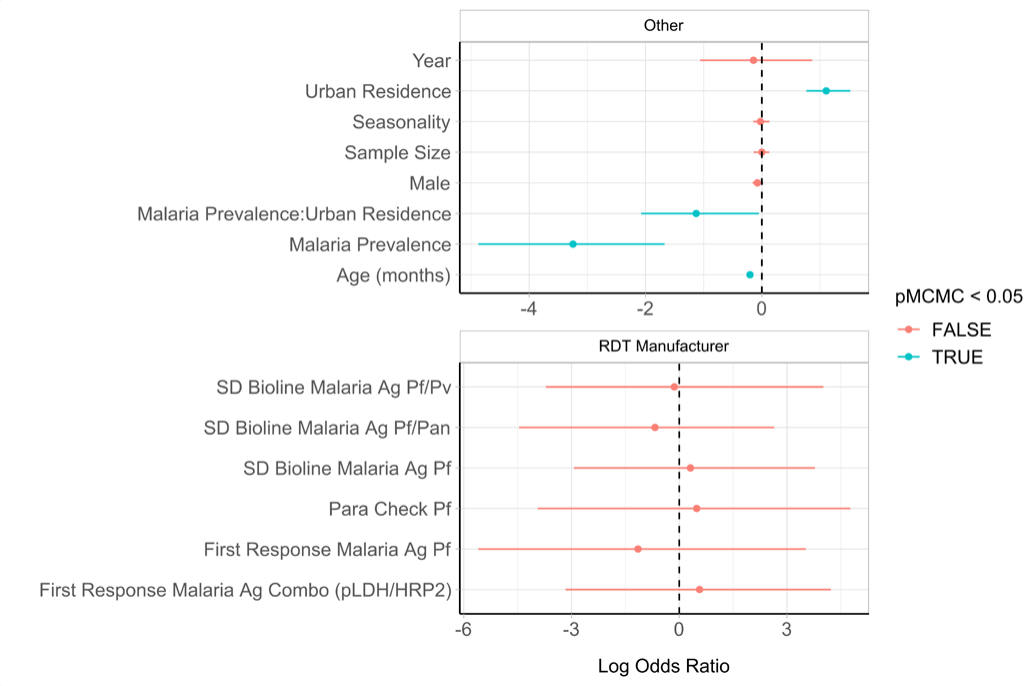
Hierarchical analysis of covariates associated with an increased risk of a false-negative rapid diagnostic test (FN-RDT) result. The log ORs for each covariate are shown with their 95% CIs as whiskers surrounding each point for (A) cluster-level and individual-level covariates and (B) RDT brand. Log ORs significantly not equal to 1 (probability of the posteriors including zero (pMCMC) <0.05) are shown in blue and were observed for the type of residence (urban vs rural), the age of the individual, the prevalence of malaria within a cluster and the interaction between malaria prevalence and residence type. The reference for the log ORs associated with brand is CareStart Malaria HRP2/pLDH Combo.

**Figure 5:**
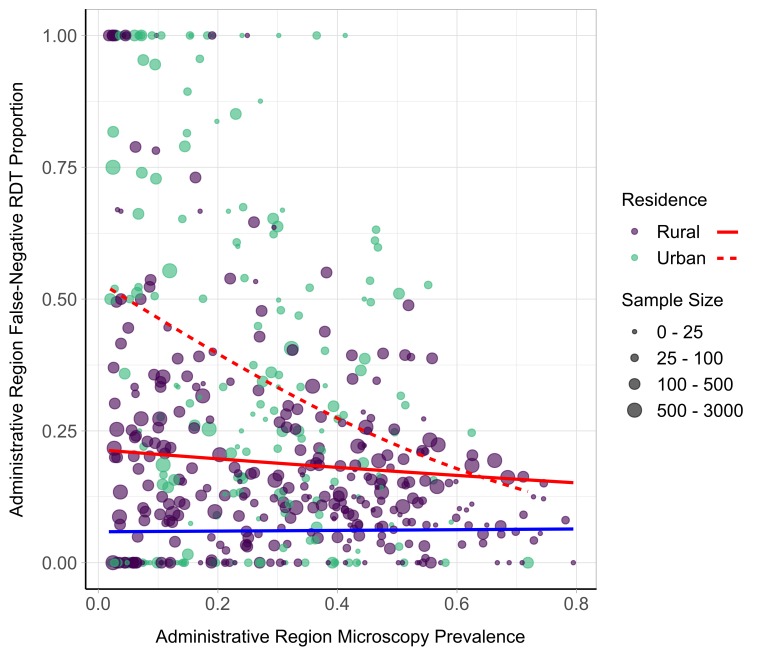
Observed relationship between proportion of false-negative rapid diagnostic test (FN-RDT) results and malaria prevalence. The weighted proportion of FN-RDT results at the first administrative region is shown on the y-axis and the weighted malaria prevalence by microscopy on the x-axis. The sample size for each region is indicated by the point size, and the generalised linear model (GLM) relationships with a binomial error structure are displayed as red curves. The relationship is further stratified by residence type, with the relationship within rural and urban areas shown with a solid and dashed line, respectively. The blue curve depicts the expected proportion of FN-RDT results based on WHO product testing results.

We observed differences in performance by RDT brand (figure 3). Seven different types of RDTs were used across the 19 surveys. The adjusted percentage of FN-RDT results ranged widely for some RDT brands, with CareStart Malaria HRP2/pLDH Combo (Access Bio, Somerset, NJ, USA) exhibiting the largest variance across administrative regions (13.7%). However, the hierarchal analysis did not identify a significant risk of an FN-RDT result associated with any RDT brand (figure 4). Three factors were found to be significantly associated with an increased risk of an FN-RDT result: lower malaria prevalence, younger age of individuals sampled and urban residence. These factors remained statistically significant when testing for interaction effects through the inclusion of a three-way interaction term. The resultant best fitting model included an additional significant interaction between malaria prevalence and urban residence (online supplementary table S4), indicating a greater increase in the risk of an FN-RDT result as prevalence decreases in urban areas. The association between younger age and FN-RDT results was driven primarily by children younger than 1 year of age (online supplementary figure S1). The impact of malaria prevalence and urban residence was further investigated by comparing the relationship between the percentage of FN-RDT results and malaria prevalence to the expected percentage of FN-RDT based on product testing results (figure 5). The proportion of FN-RDT results increased both at lower malaria prevalence and in urban areas, a finding not explained by expected differences in RDT performance based on product testing. Additionally, the rate of increase in the proportion of FN-RDT results as malaria prevalence decreased was greater in urban areas, reflecting the significant interaction identified between malaria prevalence and urbanicity.

Using these findings, we employed mathematical modelling to estimate the percentage of malaria infections that would be missed during community-based malaria surveys based on RDT results in these countries. We predict that 19.7% (10.5–29.4) of malaria infections would be missed due to FN-RDT results in the 19 countries analysed during the years surveyed (online supplementary table S5). The estimated percentage of infections missed in each country is similar to the estimates calculated directly from the surveys (online supplementary table S2), with Benin again predicted to yield the highest percentage of missed infections due to FN-RDT results (51.8% (40.2–63.4)). Most countries were predicted to have a lower percentage of missed infections compared with estimates calculated directly from the surveys, reflecting the higher proportion of FN-RDT results observed in administrative regions with lower transmission intensities. Reciprocally, countries with administrative regions exhibiting both a high malaria prevalence and a high proportion of FN-RDT results, such as Nigeria, were estimated to have a higher percentage of missed infections (27.0% (10.9–44.1)) compared with estimates calculated directly from the surveys (19.6% (16.2–23.0)). These country-wide estimates extend the survey findings and reflect the observed FN-RDT results, and the country’s prevalence of malaria, the population density in administrative regions, the historic transmission intensity and the subsequent impact on the parasitaemia of asymptomatic individuals.

## Discussion

We explored the prevalence of and covariates associated with FN-RDT results in large-scale malaria surveys in sub-Saharan Africa. We found that FN-RDT results were common in these national surveys, despite high-quality microscopy and RDT implementation. The prevalence of FN-RDTs was spatially heterogeneous, occurring most frequently in regions of low prevalence and in urban areas. We leveraged data from DHS/MIS field surveys and the WHO Product Testing Programme, which provide ongoing, high-quality assessments of malaria epidemiology and RDT performance, respectively. Our analysis provides insight into the factors associated with RDT performance in the field and confirms that FN-RDT results are not predicted by product testing alone. These findings underscore the need for careful interpretation of RDT-based prevalence estimates and ongoing efforts to improve the performance of malaria diagnostics for community surveillance.

When considered in aggregate, the effect of FN-RDT results (RDT negative, microscopy positive) on prevalence estimates may be partly counterbalanced by RDT-positive, microscopy-negative results due to lingering PfHRP2 antigenaemia after parasite clearance.^3^ The generally higher prevalence of malaria by RDT compared with microscopy observed in the present analysis is consistent with this idea. However, our findings that FN-RDT results are more common (A) in the field than in product testing and (B) in urban and low-transmission settings are relevant to design of large malaria surveys. For example, programmes might consider validating at least a subset of RDT results using an alternative diagnostic methodology (eg, PCR or microscopy), especially in urban or low-transmission areas.

The association between FN-RDT results and malaria prevalence also has potential ramifications for the roll-out of next-generation malaria diagnostics, including more highly sensitive RDTs. We observed higher FN-RDT results in regions of low malaria prevalence, a finding supported by a recent analysis of DHS and MIS data from the Democratic Republic of the Congo, Uganda and Kenya that estimated malaria prevalence and evaluated RDT performance using a Bayesian framework.^33^ The study’s results for predicted prevalence and RDT diagnostic sensitivity had a similar association to those observed in our analysis, with the highest FN-RDT results in the nation with the lowest prevalence (Kenya). The most likely explanation for this association is the increased observation of infections with lower parasite densities in areas of lower malaria prevalence.^22 34^ Similarly, our finding that FN-RDT results were more prevalent in urban compared with rural areas may also be driven by lower parasite densities, as supported by evidence suggesting that individuals in urban areas present with lower parasite densities than those in rural areas at similar transmission intensities.^35^ These findings together suggest a potential niche for improved diagnostics (eg, PCR or high-sensitivity RDTs) in community surveys conducted in both lower malaria prevalence and urban settings. It is important to emphasise that these findings cannot be applied to case management, as FN-RDT results are expected to be less common in symptomatic individuals presenting with higher parasite densities.

Our rationale for using a transmission model was foremost to extend the observed results from MIS and DHS surveys, which sample from young children in discrete clusters and demographic groups, into estimates for the whole population. For example, the model predicts that individuals older than 5 years of age are likely to exhibit lower parasite densities due to increased exposure-acquired immunity. While our analysis of MIS and DHS results was limited to children younger than 5 years old, the model provided an opportunity to estimate FN-RDT results among older individuals. Due to their decreased parasite densities, infected individuals older than 5 years of age are less likely to have FN-RDT results in the model. Along these same lines, the transmission model assumes that individuals who are symptomatic will always be detected by microscopy due to the high parasite densities associated with clinical symptoms. Thus, the modelling exercise restricts our analysis to asymptomatic infections. Although the use of the transmission model allows us to estimate the infection and immunity status of the population at risk in the countries analysed, the assumptions in the model about the detectability of asymptomatic infections and the proportion of subpatent infections are simplified. For example, the model does not directly include within-host parasitaemia and, as such, the timing of intraerythrocytic stages is not explicitly modelled, which other models have included for more accurate estimations of the submicroscopic reservoir.^36^

While we observed differences in the proportion of FN-RDT results by brand, these differences in performance were not statistically significant. Ongoing WHO product testing confirms differences in RDT performance during standardised testing, and it is unsurprising that there was variation in performance by brand and lot in the field. RDT-specific differences are likely driven by multiple factors, including the stability in different storage conditions and the avidity of different monoclonal antibodies to common HRP2 epitopes in a specific geographical region, although an association between *pfhrp2* and *pfhrp3* gene structure and RDT detection was not observed in a prior study.^37^ In addition, the ease of detecting positive test bands has also been reported to be an issue, especially among RDT brands known to produce faint test bands.^38^ As per WHO recommendations, faint bands on RDTs should be interpreted as a positive malaria result^39^; however, some evidence suggests that these bands are sometimes too faded to be seen in poor lighting.^38^ This could be one explanation for the increase in FN-RDT results observed in urban areas, where RDT results may be more likely to be assessed indoors. In addition, previous studies have shown that these faint bands are observed more often when subjects have lower parasite densities.^38^ We also found that FN-RDT results were associated with younger age, a finding driven primarily by a high proportion of FN-RDT results in subjects younger than 1 year of age (online supplementary figure S1). There are several plausible explanations for this observation, including decreased parasite densities during infancy and/or maternal anti-HRP2 antibodies^40^; a phenomenon that has not been studied to our knowledge.

There are a number of limitations to our study. First, we assume that microscopy-positive and RDT-negative results reflect a true-positive infection. We chose this approach because rigorous quality control procedures are employed for microscopy in DHS and MIS studies. Poor specificity in microscopy can occur due to a number of reasons, including poor blood film preparation, poor quality reagents and variability in both operator training and workload.^41 42^ While these factors are impossible to mitigate completely during any field study, the microscopy protocols employed during DHS and MIS studies should minimise their impact. Confirmation by PCR would provide a more sensitive assessment of parasitaemia (although PCR itself is imperfect and results vary between laboratories),^43^ but this is not typically employed in national surveys due to cost. Alternatively, confirmation of RDT findings could be achieved by measuring antigen concentrations from dried blood spots, which would enable the sensitivity of the RDT to be assessed directly.^6^ Occurrences of microscopy-positive and RDT-negative infections that are, in fact, true-negative infections will impact the estimated FN-RDT results. As a result, the modelled estimates of the percentage of infections missed during community-based surveys most likely represent an upper estimate. However, our estimates are based on the assumption that an asymptomatic individual’s probability of detection by microscopy is dependent on the prevalence of malaria (online supplementary figure S2). Consequently, the occurrence of microscopy false-positive results would also result in an increase in the estimated malaria prevalence and skew the modelled estimates. The presence of microscopy-positive and RDT-negative infections that are true-negative infections is unlikely to fully explain the observed patterns in FN-RDT with respect to malaria prevalence. For example, in Benin, which had the highest proportion of FN-RDT results, an increase in FN-RDT results was still observed in lower transmission settings. While the presence of microscopy-positive and RDT-negative infections that are true-negative infections will impact the accuracy of our FN-RDT prevalence estimates, it does not fully explain the patterns observed in the data.

Second, because speciation data were unavailable, we were unable to delineate the impact of FN-RDT results by *Plasmodium* species. As such, a portion of the observed FN-RDT results in surveys that used *P. falciparum*-specific RDTs can be attributed to infection by non-falciparum species. However, the broadly similar proportion of FN-RDT results between surveys that used *P. falciparum-*specific PfHRP2-based RDTs versus combination PfHRP2 plus pan-species lactate dehydrogenase RDTs suggests the impact of non-falciparum species on our findings is small. We also observed consistent trends in the proportion of FN-RDT results when comparing across countries with different prevalences of non-falciparum species (online supplementary figure S4).

Third, we paired the RDTs employed during DHS and MIS surveys with WHO product testing data based on RDT expiration dates, available in the published lot testing reports, rather than specific lot numbers. While the PDS score is not intended to predict FN-RDT results, it allows for an estimate of RDT performance in the field based on the assumed distribution of parasite densities of infections detectable by microscopy. Analysis of qPCR parasite densities among microscopy-positive subjects enrolled in other cross-sectional studies suggests that the differences between the expected FN-RDT prevalence based on product testing and the observed FN-RDT prevalence in DHS/MIS surveys were not driven simply by parasite densities below the RDTs’ LOD. Fourth, we did not have data on differences in RDT storage conditions, the quality of operator use, or supervision across the DHS and MIS surveys. However, their protocols and rigorous training procedures suggest that RDTs were deployed using best practices. In addition, the survey year was not a risk factor for FN-RDT results, which provides some evidence that operator use was consistent throughout included studies.

Finally, we do not have data on the prevalence of *pfhrp2/3* gene deletions in most of the countries. Although there is some correlation between the countries predicted to be at the highest risk for *pfhrp2/3* gene deletions by recent modelling and those with the highest FN-RDT results,^13^ the contribution of *pfhrp2/3* gene deletions to the FN-RDT results in our study cannot be determined definitively. We suspect that other factors are major drivers of the observed FN-RDT distribution. Indeed, FN-RDT results were common in Mozambique, where a recent study demonstrated that only 1.45% of parasites had *pfhrp2/3* deletions.^44^ In addition, the insignificant impact of seasonality in the hierarchical model argues against a major role for *pfhrp2/3* deletions in driving FN-RDT results in included studies, based on recent modelling suggesting that FN-RDT results due to *pfhrp2/3* deletions are more common at the beginning of a transmission season when monoclonal infections are often more prevalent.^45^

The results presented here demonstrate that FN-RDT results are common in community malaria surveys throughout sub-Saharan Africa. Our findings confirm that RDT performance in field settings cannot be predicted by lot testing alone and indicate that FN-RDT results are more common in low-transmission and urban settings. To complement surveillance of RDT brand and lot performance, continued field effectiveness studies are required. Additionally, our findings underscore the need for thoughtful deployment of next-generation, highly sensitive RDTs and ongoing efforts to improve malaria diagnostics.
