# Supplementary material for: False-negative malaria rapid diagnostic test results and their impact on community-based malaria surveys in sub-Saharan Africa
Source: BMJ Glob Health. 2019 Jul 29;4(4):e001582. doi: 10.1136/bmjgh-2019-001582 (PMC6666813; doi:10.1136/bmjgh-2019-001582)
Supplement: Supplementary data [file bmjgh-2019-001582supp001.pdf]

False-negative malaria rapid diagnostic test results and their impact on community-based malaria surveys in sub-Saharan Africa

SUPPLEMENTARY INFORMATION

Watson OJ,[*†1] Sumner KM,[*2] Janko M,[3] Goel V,[4] Winskill P,[1] Slater HC,[1] Ghani AG,[1] Meshnick SR,[2] Parr JB[5]

Affiliations:

1. MRC Centre for Global Infectious Disease Analysis, Department of Infectious Disease Epidemiology, Imperial College London
2. Department of Epidemiology, Gillings School for Global Public Health, University of North Carolina, Chapel Hill, NC 27599, USA
3. Duke Global Health Institute, Duke University, Durham, NC 27710, USA
4. Department of Geography, University of North Carolina, Chapel Hill, NC 27599, USA
5. Division of Infectious Diseases, Department of Medicine, School of Medicine, University of North Carolina, Chapel Hill, NC 27599, USA

* Contributed Equally

[†] **Corresponding Author:**
Mr Oliver Watson, MRC Centre for Outbreak Analysis and Modelling, Department of Infectious Disease Epidemiology, Imperial College London, London W2 1PG, UK : o.watson15@imperial.ac.uk

Table S1: Published studies reporting P. falciparum pfhrp2 and/or pfhrp3 deletions in sub-Saharan Africa

Country	Reference
Ghana	Amoah, L.E. et al. Plasmodium falciparum histidine rich protein-2 diversity and the implications for PfHRP2: based malaria rapid diagnostic tests in Ghana. Malar Journal 2016; **15**(101).<br>Owusu, E. D. A. et al. Plasmodium falciparum diagnostic tools in HIV-positive under-5-year-olds in two ART clinics in Ghana: are there missed infections? Malaria Journal, 2018; **17**(92).
Angola	Plucinski, M.M. et al. Malaria Parasite Density in Individuals with Different Rapid Diagnostic Test Results and Concentrations of HRP2 Antigen. Am. J. Trop. Med. Hyg. 2019; **100**(5): 1202–1203.
Eritrea	Menegon M, et al. Identification of Plasmodium falciparum isolates lacking histidine-rich protein 2 and 3 in Eritrea. Infect Genet Evol 2017; **55**: 131–4.<br><br>Berhane, A. et al. Major Threat to Malaria Control Programs by Plasmodium falciparum Lacking Histidine-Rich Protein 2, Eritrea. Emerging Infectious Diseases, 2018; **24**(3): 462–470.
Kenya	Beshir, K.B. et al. Plasmodium falciparum parasites with histidine-rich protein 2 (pfhrp2) and pfhrp3 gene deletions in two endemic regions of Kenya. Scientific Reports 2017; **7**(1): 1–10.
Mozambique	Gupta, H. et al. Molecular surveillance of pfhrp2 and pfhrp3 deletions in Plasmodium falciparum isolates from Mozambique. Malaria Journal 2017; **16**(1): 1–7.
Mali	Koita, O.A. et al. False-Negative Rapid Diagnostic Tests for Malaria and Deletion of the Histidine-Rich Repeat Region of the hrp2 Gene. Am. J. Trop. Med. Hyg. 2012; **86**(2): 194–198.
Rwanda	Kozycki, C.T. et al. False-negative malaria rapid diagnostic tests in Rwanda: impact of Plasmodium falciparum isolates lacking hrp2 and declining malaria transmission. Malaria Journal 2017; **16**(123).
Zambia	Laban, N.M. et al. Comparison of a PfHRP2-based rapid diagnostic test and PCR for malaria in a low prevalence setting in rural southern Zambia: implications for elimination. Malaria Journal 2015; **14**(25).<br><br>Kobayashi, T. et al. The Search for Plasmodium falciparum histidine-rich protein 2/3 Deletions in Zambia and Implications for Plasmodium falciparum histidine-rich protein 2-Based Rapid Diagnostic Tests. Am J Trop Med Hyg. 2019; **100**(4): 842-845.
Uganda	Nsobya, S. et al. Deletions of pfhrp2 and pfhrp3 in RDT-negative Plasmodium falciparum isolates from Uganda (unpublished). American Society of Tropical Medicine and Hygiene 65th Annual Conference 2016.
Democratic Republic of Congo	Parr, J.B. et al. Pfhrp2-deleted Plasmodium falciparum parasites in the Democratic Republic of Congo: A national cross-sectional survey. Journal of Infectious Diseases 2016; **216**(1): 36–44.
Senegal	Wurtz, N. et al. Pfhrp2 and pfhrp3 polymorphisms in Plasmodium falciparum isolates from Dakar, Senegal: impact on rapid malaria diagnostic tests. Malaria Journal, 2013; **12**(34).
Swaziland	Ranadive, N. et al. Limitations of rapid diagnostic testing in patients with suspected malaria: A diagnostic accuracy evaluation from Swaziland, a low-endemicity country aiming for malaria elimination. Clinical Infectious Diseases 2017. **64**(9): 1221–1227.

Table S2: Characteristics of study subjects.

Characteristic	Subjects	Microscopy+		RDT+		Microscopy+/RDT-	
	n	n	Prevalence, %*	n	Prevalence, %*	n	Prevalence, %*
**Residence**							
Rural	63 530	17 742	28.2 [27.1,29.2]	22 329	34.7 [33.5,35.8]	3 160	18.0 [17.0,19.0]
Urban	21 470	2 647	12.1 [10.9,13.2]	3 503	16.1 [14.5,17.7]	893	34.0 [30.3,37.6]
**Brand**							
CareStart Malaria HRP2/pLDH Combo, G0131	5 357	349	9.2 [6.7,11.6]	399	10.2 [7.8,12.5]	73	23.6 [18.0,29.1]
First Response (FR) Malaria Ag Pf, I13FRC30	3 185	9 79	26.6 [23.1,30.1]	2 984	30.2 [27.9,32.4]	88	10.1 [7.5,12.6]
FR Malaria Ag Combo (pLDH/HRP2), I16FRC	9 885	2 686	26.8 [24.7,29.0]	1 635	44.7 [40.3,49.1]	619	22.9 [20.6,25.2]
Para Check Pf, 30301025/30301025	12 917	4 794	38.0 [35.5,40.5]	5 543	43.7 [41.1,46.3]	1 146	23.3 [21.0,25.6]
SD Bioline Malaria Ag Pf, 05FK50/05FK53	24 388	8 427	33.9 [32.2,35.5]	11 084	43.3 [41.5,45.2]	1 540	18.2 [16.8,19.6]
SD Bioline Malaria Ag Pf/Pan, 05FK60/05FK66	20 940	1 354	6.3 [5.5,7.3]	2 098	10.1 [8.9,11.3]	231	17.1 [14.3,19.9]
SD Bioline Malaria Ag Pf/Pv, 05FK80	8 328	1 800	24.6 [22.2,27.1]	2 089	27.8 [25.1,30.4]	356	19.5 [16.9,22.1]
**Country, year**							
Angola, 2011	3 430	335	10.1 [7.2,13.1]	432	13.5 [9.6,17.4]	70	19.3 [13.8,25.2]
Benin, 2011-12	3 810	1 117	28.1 [25.9,30.3]	1 019	24.6 [22.5,26.8]	577	54.1 [50.0,58.2]
Burkina Faso, 2014	6 152	2 914	45.7 [42.1,49.3]	3 964	61.3 [57.8,64.8]	377	13.7 [11.8,15.5]
Burundi, 2012	3 718	601	17.3 [13.6,21.1]	764	22.0 [17.5,26.5]	53	9.4 [6.2,12.5]
Côte D'Ivoire, 2012	3 346	570	17.4 [14.6,20.3]	1 508	41.4 [37.7,45.0]	110	23.1 [17.9,28.4]
Democratic Republic of The Congo, 2013-14	8 216	2 146	22.2 [19.7,24.7]	2 958	30.9 [27.6,34.2]	399	18.2 [15.1,21.4]
Guinea, 2012	3 215	1 398	44.1 [40.0,48.1]	1 465	46.8 [42.4,51.3]	380	25.9 [22.2,29.5]
Kenya, 2015	3 432	180	5.0 [3.4,6.5]	318	9.0 [6.2,11.8]	30	17.7 [11.3,24.0]
Liberia, 2011	3 185	979	26.6 [23.1,30.1]	1 635	44.7 [40.3,49.1]	88	10.1 [7.5,12.6]
Madagascar, 2013	5 357	349	9.2 [6.7,11.6]	399	10.2 [7.8,12.5]	73	23.6 [18.0,29.1]
Malawi, 2014	1 935	512	33.0 [25.7,40.2]	578	36.3 [29.3,43.3]	71	11.2 [6.9,15.5]
Mali, 2012-2013	4 739	2 285	51.0 [47.6,54.3]	2 076	47.0 [43.6,50.4]	583	24.0 [21.1,27.0]
Mozambique, 2011	4 898	1 465	35.0 [31.6,38.4]	1 657	38.0 [34.6,41.3]	286	19.5 [16.7,22.4]
Nigeria, 2010	5 148	1 954	41.8 [37.4,46.3]	2 427	51.4 [47.0,55.9]	429	19.6 [16.2,23.0]
Rwanda, 2014-2015	3 455	76	2.3 [1.7,3.0]	259	7.9 [6.4,9.4]	9	11.4 [3.9,19.0]
Senegal, 2012-13	6 085	220	2.8 [1.8,3.7]	254	3.3 [2.2,4.5]	64	29.4 [20.7,38.1]
Togo, 2013-14	3 215	1 212	36.6 [33.0,40.3]	1 260	38.0 [34.1,42.0]	230	19.8 [16.6,23.1]
Uganda, 2009	3 959	1 723	42.5 [37.3,47.7]	2 097	51.9 [46.4,57.4]	140	8.4 [6.6,10.2]
United Republic Of Tanzania, 2011-12	7 705	353	4.0 [3.1,4.9]	762	9.3 [7.7,11.0]	84	27.5 [20.7,34.2]
Total	85 000	20 389	24.4 [23.5,25.2]	25 832	30.3 [29.4,31.2]	4 053	19.9 [19.0,20.9]

*** Weighted Prevalence [95% CI]**

Table S3: Spatial Clustering Analysis. Spatial autocorrelation was assessed within each country using Global Moran's I. P-values were calculated by running 999 permutations of Monte Carlo simulations. For each country's neighbourhood weight matrix, the arc distance radius was chosen using correlograms of the FN-RDT results. The distance radius was chosen to ensure each DHS cluster had at least one neighbouring value.

Country	Arc Distance (km) Weight	Global Moran's I (p-value)
Angola	130.489	0.196 (0.009)
Benin	41.042	0.134 (0.001)
Burkina Faso	57.215	0.104 (0.006)
Burundi	18.602	0.161 (0.030)
Democratic Republic of Congo	211.424	0.035 (0.027)
Cote D'Ivoire	76.095	0.087 (0.006)
Guinea	68.344	0.069 (0.009)
Kenya	164.892	0.022 (0.143)
Liberia	69.701	-0.014 (0.448)
Madagascar	99.683	-0.063 (0.202)
Malawi	54.594	0.069 (0.091)
Mali	70.447	0.216 (0.001)
Mozambique	101.692	0.179 (0.001)
Nigeria	193.082	0.053 (0.004)
Rwanda	15.190	-0.049 (0.317)
Senegal	72.609	-0.054 (0.360)
Togo	25.387	0.061 (0.035)
Uganda	64.696	0.051 (0.083)
United Republic of Tanzania	137.902	-0.001 (0.418)

Table S4: Hierarchical model selection. Interactions between significant terms identified in the model with no interactions were explored and the best fitting model identified via AICc comparisons.

Model	Degrees of Freedom	AICc	ΔAICc	Log Likelihood
Prevalence:Residence	19	13840.02	0	-6900.99
No Interaction	18	13846.77	6.745915	-6905.37
Age:Residence	19	13854.16	14.13779	-6908.06
Prevalence:Age	19	13854.35	14.3239	-6908.16
3-way Interaction	22	13883.09	43.06817	-6919.52

Table S5: Estimates of missed infections within country-wide surveys due to false-negative RDT results. Estimates are generated for the survey years described in Table S2**.**

**Country**	**% Missed Infections**	**95% Confidence Interval**
Angola	24.6	11.6 – 38.2
Benin	51.8	40.2 – 63.4
Burkina Faso	12.8	7.7 – 18.0
Burundi	8.6	2.1 – 15.3
Côte d'Ivoire	24.9	10.9 – 40.0
Democratic Republic of the Congo	17.2	9.2 – 25.4
Guinea	25.1	16.7 – 33.5
Kenya	19.6	7.4 – 32.7
Liberia	9.6	2.5 – 17.5
Madagascar	22.8	13.0 – 34.3
Malawi	10.6	4.0 – 17.1
Mali	24.2	18.1 – 30.5
Mozambique	19.9	12.8 – 27.0
Nigeria	27.1	10.9 – 44.1
Rwanda	12.2	1.0 – 27.7
Senegal	40.9	20.1 – 62.3
Togo	20.0	14.0 – 25.9
Uganda	8.6	5.1 – 13.2
United Republic of Tanzania	36.4	22.2 – 51.8
**Total**	**19.7**	**10.5 – 29.4**

Figure S1: Observed relationship between the proportion of false-negative (FN-RDT) results and the age of individuals tested. The weighted proportion of FN-RDT results is shown on the y-axis and the age of individuals on the x-axis, with 12 months indicated by the dashed line. The sample size for each age is indicated by the point size, and a locally weighted regression line is shown in blue.

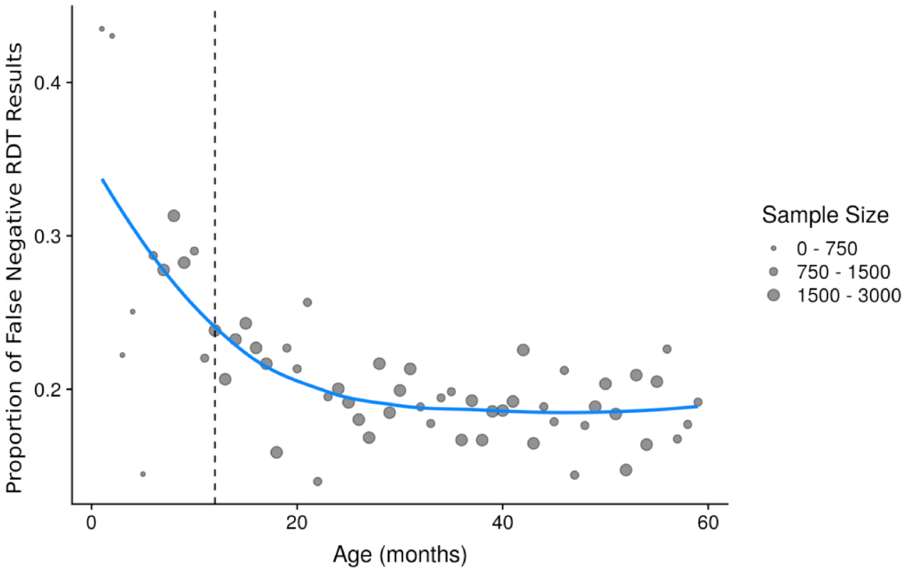

Figure S2: Observed relationship within the Imperial transmission model between the proportion of asymptomatic infections (states A and U) that will be detected by microscopy and prevalence of malaria by microscopy within the administrative regions analysed. A locally weighted regression line is shown in blue with the confidence interval indicated in grey.

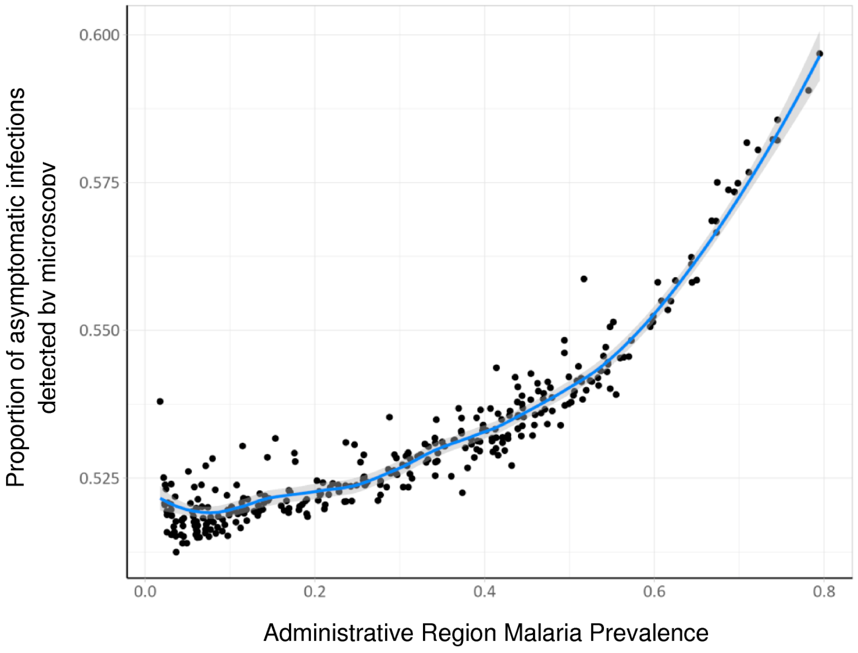

Figure S3: Observed parasite densities of microscopy-positive infections in other cross-sectional studies. Boxplots show the parasite densities (parasites per µL) as determined by qPCR among infections identified in cross-sectional studies from countries included in the present analysis. Each infection was also detected by microscopy. The vertical dashed line at 200p/µL is included for reference to the WHO Product Testing programme's Panel Detection Score (PDS). The country and reference number is shown on the y axis.

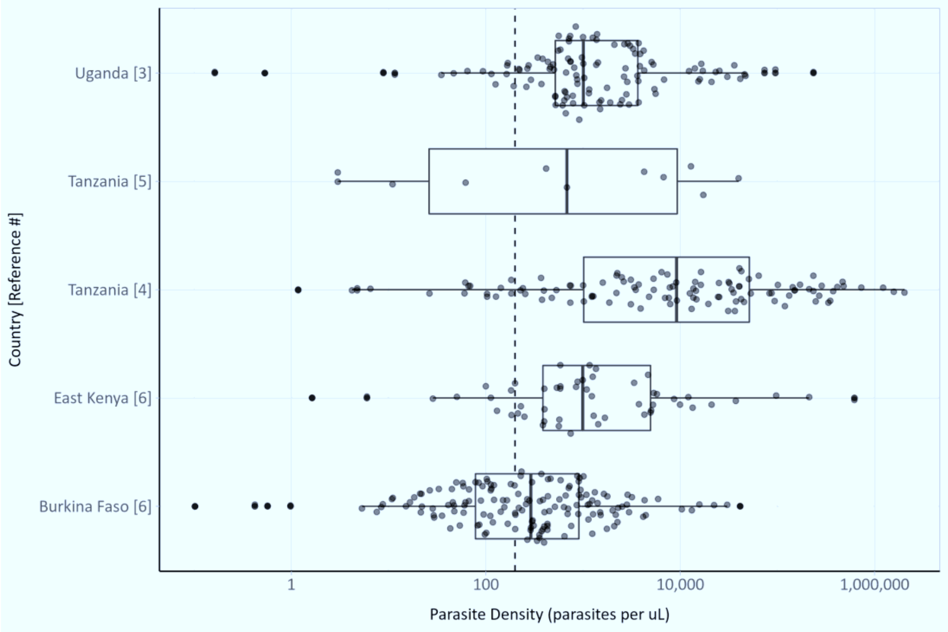

Figure S4: Observed relationship between the proportion of false-negative rapid diagnostic test (FN-RDT) results and malaria prevalence at country levels. The weighted proportion of FN-RDT results at the first administrative region is shown on the y-axis and the weighted malaria prevalence by microscopy on the x-axis. The sample size for each region is indicated by the point size, and the GLM relationship with a binomial error structure shown for each country in blue.

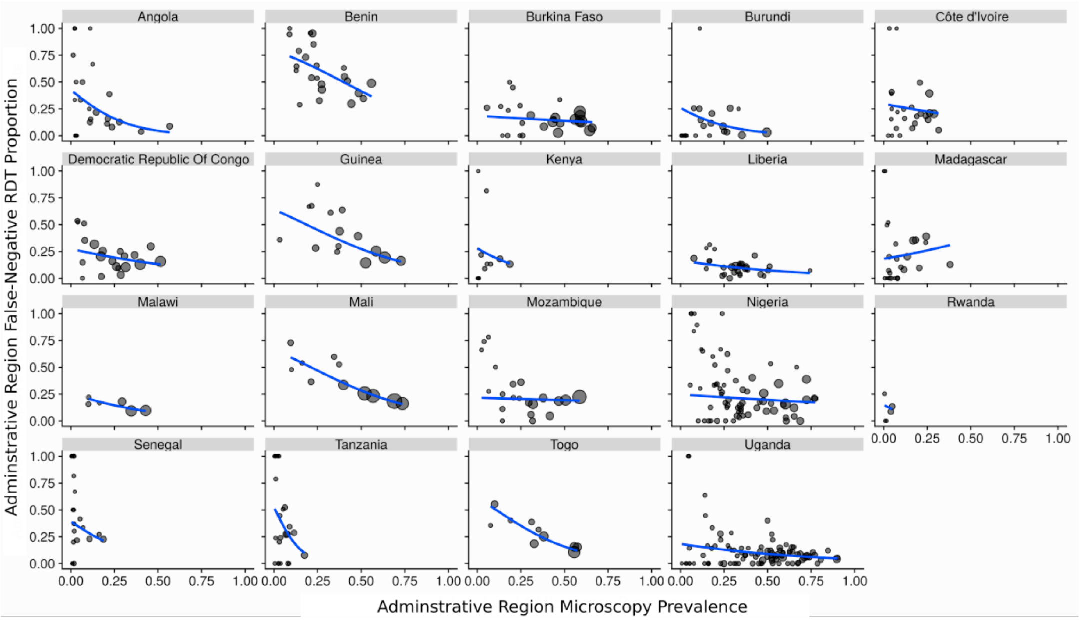

Additional methodology for derivation of p(detection)

Within the Imperial College transmission model, people are considered to exist in one of six infection states (Figure S3): susceptible (S), clinical disease (D), clinically diseased and receiving treatment (T), potentially patent asymptomatic infection (A), subpatent asymptomatic infection (U) and in a protective state of prophylaxis (P). A subpatent infection, state U, is an asymptomatic infection that will never be detected by microscopy due to low parasite densities resulting from nearing the end of their infection course. Individuals in state A are also asymptomatic infections, however, they are in the earlier stages of their asymptomatic infection and consequently have higher parasite densities that may be detected by microscopy. The probability that individuals in state A will be detected, however, depends on their acquired blood stage immunity, which decreases the parasite density of an asymptomatic infection.

Figure S3: Human component of the Imperial College Transmission model. Dashed arrows show superinfection events.

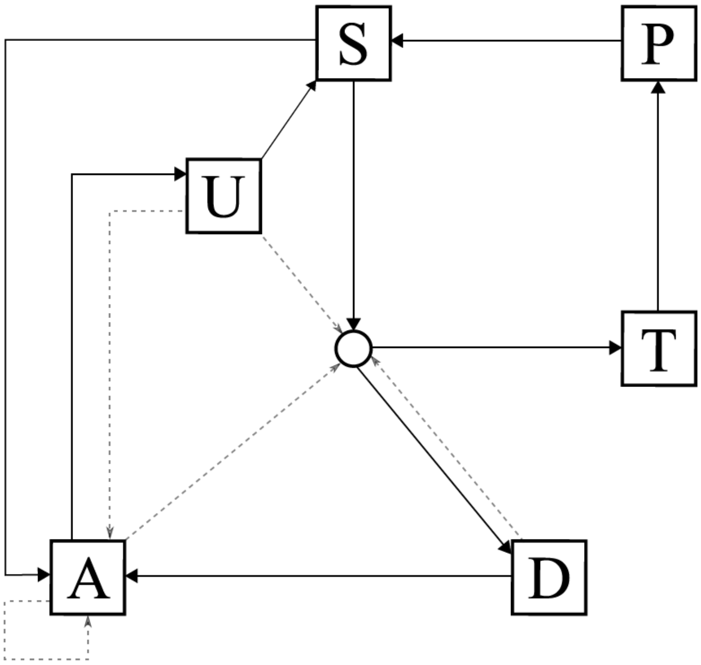

The probability that individual i will be detected by microscopy depends on their infection state, acquired blood stage immunity and their age:

Infection State	p(detection)
S	0
P	0
T	1
D	1
U	0
A	q_i

q_i is an individual's probability of being detected by microscopy when in state A. q_i is given by:

$$q_i = d_1 + \left(\frac{1 - d_1}{1 + \left(\frac{I_{D_i}}{I_{D0}} \right)^{\kappa_D} f_D} \right)$$

where I_{D_i} is an individual's exposure acquired blood stage immunity, which both reduces the probability of detection and infectiousness to mosquitoes, d_1 is the minimum probability of detection due to maximum immunity, and I_{D0} and κ_D are scale and shape parameters respectively. f_D is dependent only on an individual's age is given by:

$$\frac{df_D}{da} = 1 - \frac{1 - f_{D0}}{1 + \left(\frac{a}{a_D} \right)^{\gamma_D}}$$

where f_{D0} represents the time-scale at which immunity changes with age, a, and a_D and γ_D are scale and shape parameters respectively. See Winskill et al (2017)[1] for full model specification and parameter definitions.[1] See Griffin et al (2014)[2] for the specific parameter fitting of these parameters.

Therefore, the probability that an individual in state A will produce a positive test result by microscopy will decrease at higher transmission intensities due to the resultant higher acquired blood stage immunity. However, this is offset by the higher transmission intensity leading to an increased ratio of individuals in state A rather than state U, which increases the chances that any given asymptomatic individual (states A and U) will yield a positive microscopy result.

Parasite Densities of asymptomatic infections detected by microscopy

To assess the validity of using the 200p/µL PDS result to adjust the observed FN-RDT results, we sourced data collected from the countries included within our study that recorded parasite densities and the detectability by microscopy of asymptomatic individuals. This search yielded five studies,[3–6] which were used to describe the distribution of parasite densities likely to be found within the samples collected within our study. This data is presented within Supplementary Figure S3 and shows that the majority of asymptomatic infections that are detected by microscopy would have parasite densities greater than 200p/µL.

References

1 Winskill P, Slater HC, Griffin JT, Ghani AC, Walker PGT. The US President's Malaria Initiative, Plasmodium falciparum transmission and mortality: A modelling study. PLoS Med 2017; **14**: 1–14.

2 Griffin JT, Ferguson NM, Ghani AC. Estimates of the changing age-burden of Plasmodium falciparum malaria disease in sub-Saharan Africa. Nat Commun 2014; **5**: 3136.

3 Das S, Jang IK, Barney B, et al. Performance of a high-sensitivity rapid diagnostic test for Plasmodium falciparum malaria in asymptomatic individuals from Uganda and Myanmar and naive human challenge infections. Am J Trop Med Hyg 2017; **97**: 1540–50.

4 Hofmann N, Mwingira F, Shekalaghe S, Robinson LJ, Mueller I, Felger I. Ultra-Sensitive Detection of Plasmodium falciparum by Amplification of Multi-Copy Subtelomeric Targets. PLoS Med 2015; **12**. DOI:10.1371/journal.pmed.1001788.

5 Gonçalves BP, Kapulu MC, Sawa P, et al. Examining the human infectious reservoir for Plasmodium falciparum malaria in areas of differing transmission intensity. Nat Commun 2017; **8**. DOI:10.1038/s41467-017-01270-4.

6 Mwingira F, Genton B, Kabanywanyi A-NM, Felger I. Comparison of detection methods to estimate asexual Plasmodium falciparum parasite prevalence and gametocyte carriage in a community survey in Tanzania. Malar J 2014; **13**: 1–8.
